# Effect of Cyclic Heat Stress on Feeding-Related Hypothalamic Neuropeptides of Three Broiler Populations and Their Ancestor Jungle Fowl

**DOI:** 10.3389/fphys.2021.809341

**Published:** 2021-12-23

**Authors:** Giorgio Brugaletta, Elizabeth Greene, Travis Tabler, Sara Orlowski, Federico Sirri, Sami Dridi

**Affiliations:** ^1^Department of Agricultural and Food Sciences, Alma Mater Studiorum – University of Bologna, Bologna, Italy; ^2^Center of Excellence for Poultry Science, University of Arkansas, Fayetteville, AR, United States

**Keywords:** broiler chicken, ancestor, heat stress, feed intake, hypothalamic neuropeptides

## Abstract

Heat stress (HS) has been increasingly jeopardizing the sustainability of the poultry production. Moreover, modern high-performing chickens are far less able to withstand HS than their predecessors due to higher growth rate and metabolic rates. Performance losses caused by HS are mainly ascribed to decreases in feed consumption. Since feed intake is tightly controlled by the hypothalamic centers of hunger and satiety, we sought to determine the effect of chronic cyclic HS on the expression of feeding-related hypothalamic neuropeptides (FRHN) in unselected chickens (i.e., the ancestor junglefowl—JF) and three broiler lines from diverse stages of genetic selection (i.e., the slow growing ACRB, the moderate growing 95RN, and the fast growing MRB). From 29 to 56 days, birds (*n* = 150 birds for each population) were subjected to either thermoneutral (TN, 25°C) or cyclic heat stress (HS, 36°C, 0900–1,800 h) conditions. Molecular data were analyzed by two-way ANOVA with interaction between the main factors, namely environmental temperature and line. The expression of major FHRN, like neuropeptide Y, agouti-related peptide, proopiomelanocortin, and cocaine and amphetamine regulated transcript remained unchanged. However, melanocortin receptor 1 exhibited a line-dependent decreasing trend from JF to MRB under both TN and HS (*p* = 0.09), adiponectin expression showed a distinct trend toward significance with 95RB exhibiting the highest mRNA level irrespective of the environmental temperature (*p* = 0.08), and JF had a greater mRNA abundance of visfatin than ACRB under TN (*p* < 0.05). The hypothalamic integration of circadian information, acclimation to long-lasting HS exposure, stable hypothalamic pathways unaffected by evolution and genetic selection, focus on mRNA abundances, and use of the entire hypothalamus masking gene expression in specific hypothalamic nuclei are all possible explanations for the lack of variations observed in this study. In conclusion, this is the first assessment of the impacts of heat stress on feeding-related hypothalamic neuropeptides of chicken, with a valuable and informative comparison between the ancestor junglefowl and three differently performing broiler lines.

## Introduction

Numerous factors have contributed to the formidable expansion of poultry meat production. However, genetic selection has been widely deemed to be one of the most important drivers for this incomparable success in the history of livestock industry. When it comes to broiler chicken genetic advancements, enlightening papers published by Havenstein et al., 1994a,b, 2003a,b) certainly come to mind of poultry scientists and professionals. These authors clearly showed the impressive improvement in broiler’s growth rate, feed efficiency, and yield ascribable to breeding programs carried out since the 1950s. Their findings have also been recently supported by Zuidhof et al. (2014). Tallentire et al. (2018) have even posited that the biological limits for further improvements are about to be reached. Moreover, these outstanding enhancements have come at a price in terms of animals’ resilience and welfare. For instance, modern high-performing chickens are far less able to cope with high environmental temperatures than their predecessors (Cahaner and Leenstra, 1992; Yunis and Cahaner, 1999). The reason for this greater sensitivity to hot conditions has primarily been attributed to faster growth and higher metabolic rate characterizing the currently available lines (Yahav, 2009). Even though their body has undergone selection-mediated changes, chickens still are homeothermic animals lacking sweat glands and relying on sensible heat loss—through conduction, radiation, and convection (Renaudeau et al., 2012; Rostagno, 2020)—and latent heat loss—via evaporation of water from the respiratory tract (Rajaei-Sharifabadi et al., 2017)—to thermoregulate.

Heat stress (HS) is a thermoregulatory failure resulting from a negative balance between heat dissipated and metabolic heat generated by animals. Besides being potentially lethal, HS unfavorably affects the behavior, physiology, productive potential, and well-being of chickens (Renaudeau et al., 2012; Rostagno, 2020). The voluntary reduction in feed intake is an evolutionary conserved strategy adopted by animals to reduce the thermogenesis linked to digestion, absorption, and nutrient utilization (Baumgard and Rhoads, 2013). Performance losses of heat-stressed poultry have commonly been ascribed to decreases in feed consumption (Dale and Fuller, 1980), although pair-feeding experiments have revealed that almost half of them are anorexia-unrelated (Dale and Fuller, 1980; Geraert et al., 1996; Renaudeau et al., 2012).

Given the extraordinary strides made by genetic selection, higher proneness of modern broilers to HS, and remarkable HS-caused modifications in feed intake of poultry, we hypothesized that the hypothalamus, known as a vital control center of feeding behavior and energy homeostasis (Simpson et al., 2008; Waterson and Horvath, 2015; Timper and Brüning, 2017; Luquet et al., 2019), can be differently affected in unselected chickens and genotypes from diverse breeding era stages. Hence, we sought to determine here the effect of chronic cyclic HS on the expression of feeding-related hypothalamic neuropeptides (FRHN) in three broiler populations and their common ancestor, namely the jungle fowl.

## Materials and Methods

### Chicken Populations

The four chicken lines employed in this study are maintained, bred, and hatched at the poultry facilities of the University of Arkansas. Each generation has been randomly mated except for full and half sibs in order to curtail the inbreeding. The slow-growth Athens Canadian Random Bred (ACRB) represented a commercial broiler from the 1950s (Collins et al., 2016). The moderate growing 1995 random bred (95RN), obtained from 7 parent stock male and 6 parent stock female lines, was used as representative of meat-type chickens available in the 1990s (Harford et al., 2014). The third line was the high yielding and fast growing modern random bred (MRB) conceived and developed at the University of Arkansas as a mixture of broiler hybrids currently used. The fourth populations was the ancestor of the domestic chicken, that is the South East Asian junglefowl (JF) (Gyles et al., 1967; Wall and Anthony, 1995). The present study was approved by the University of Arkansas Animal Care and Use Committee (protocols 18,083 and 16,084), and conducted in compliance with the guide for the care and use of laboratory animals of the National Institutes of Health.

### Animal Husbandry and Experimental Design

At hatch, 600 vent-sexed male chicks were individually wing-tagged with a number code, grouped by line (*n* = 150), and randomly placed in 12 environmentally controlled chambers divided in 2 equally sized pens (25 birds/pen). Each pen represented a replicate for a 2 × 4 experimental design with environmental temperature and line as main factors. Pens were equipped with a feeder and a drinker, while the floor was covered with wood shavings as bedding material. All birds were manually fed and watered *ad libitum* on a daily basis. A two-phase feeding program, based on commercially available starter (0–28 days) and finisher diets (29–56 days), was used. The artificial photoperiod was 23L:1D during the first 7 days, while 20L:4D for the remainder of the trial. The environmental temperature was progressively reduced in all chambers from 32°C (0–3 days), to 31°C (4–6 days), 29°C (7–10 days), 27°C (11–14 days), and 25°C (15–28 days). Later, from 29 to 56 days, birds were exposed to either thermoneutral (TN, 25°C) or cyclic heat stress (HS, 36°C, 0900–1,800 h) conditions. Therefore, 6 rooms were kept at TN, whereas the others cyclically subjected to HS (Table 1).

**TABLE 1:** Experimental design and assignment of lines to chambers and pens.

Chamber	Treatment	Pen	Line
1	HS	1	ACRB
		2	MRB
2	TN	3	JF
		4	MRB
3	HS	5	ACRB
		6	95RAN
4	TN	7	JF
		8	95RAN
5	HS	9	95RAN
		10	MRB
6	HS	11	ACRB
		12	JF
7	TN	13	ACRB
		14	95RAN
8	HS	15	JF
		16	MRB
9	TN	17	ACRB
		18	MRB
10	TN	19	JF
		20	ACRB
11	HS	21	JF
		22	95RAN
12	TN	23	MRB
		24	95RAN

### Productive Performance Measurement and Hypothalamic Sample Collection

Body weight (BW) and feed intake (FI) were recorded weekly. The number and BW of dead or culled birds were considered while computing the feed conversion ratio (FCR) of each replicate as previously reported (Abdelli et al., 2021). Sample collection was carried out at the end of the grow-out period (56 days) after feeding all animals and applying, in the appropriate rooms, HS for at least 2 h. Two birds per pen—i.e., 6 birds from each combination of environmental temperature and line—were randomly selected and euthanized by cervical dislocation. Hypothalamic samples were harvested and processed following the techniques illustrated by Piekarski et al. (2016). Briefly, the brain was pulled from the skull and submerged in 2-methylbutane (Sigma, St. Louis, MO) in dry ice for 60 s. This treatment preserved the brain structure and provided firmness necessary to make precise cuts for hypothalamus extraction. The hypothalamic dissection was performed according to the stereotaxic atlas of the chick brain authored by Kuenzel and Masson (1988). Brain samples were placed on a cold metal plate with the ventral side exposed for dissection. The hypothalamus was dissected with an anterior cut at the corticoseptomesencephalic tract (also known as septopalliomesencephalic tract) and a posterior cut at the third oculomotor nerve. Laterally, 2 mm from the brain midline, two cuts were performed on either side. Dorsally, a 5 mm cut from the brain base was performed to get the entire hypothalamus.

### RNA Isolation, Reverse Transcription, and Real-Time Quantitative PCR

The analytical process described in this section was conducted along the lines of previous studies published by our laboratory (Rajaei-Sharifabadi et al., 2017; Greene et al., 2019, 2020). Total RNA was isolated from the hypothalamic samples (*n* = 48) by way of Trizol reagent (Life Technologies, Carlsbad, CA) according to the recommendations provided by the manufacturer. RNA purity and concentrations were assessed via Take3 micro-volume plate and Synergy HT multimode microplate reader (BioTek, Winooski, VT). One sample belonging to JF-HS group showed poor RNA quality and, therefore, was excluded. RNA samples were RQ1 RNase-free DNase treated (Promega, Madison, WI), and 1 μg RNA was reverse transcribed by means of qScript cDNA Synthesis Kit (Quanta Biosciences, Gaithersburg, MD). The reverse transcription reaction was performed at 42°C for 30 min followed by a 5 min incubation at 85°C. Real-time quantitative PCR (Applied Biosystems 7500 Real-Time PCR System) was carried out in a total 12.5 μL reaction using 2.5 μL of cDNA, 0.5 μL of each forward and reverse specific primer, and SYBR Green Master Mix (Thermo-Fisher Scientific, Rockford, IL). The chicken-specific oligonucleotide primers used in this study are listed in Table 2. The qPCR cycling settings were 50°C for 2 min and 95°C for 10 min, followed by 40 cycles of a two-step amplification program (95°C for 15 s and 58°C for 60 s). Relative expression of target genes was calculated via the comparative *C*_*T*_ method reviewed by Schmittgen and Livak (2008), using 18S rRNA as a housekeeping gene and JF-TN group as calibrator.

**TABLE 2:** List of qPCR chicken-specific oligonucleotide primers.

Gene	Accession number^†^	Primer sequence (5’→3’)	Orientation^§^	Product size (bp)
NPY	NM_205473	CATGCAGGGCACCATGAG	F	55
		CAGCGACAAGGCGAAAGTC	R	
AgRP	AB029443	GCGGGAGCTTTCACAGAACA	F	58
		CGACAGGATTGACCCCAAAA	R	
POMC	AB019555	GCCAGACCCCGCTGATG	F	56
		CTTGTAGGCGCTTTTGACGAT	R	
CART	KC249966	GCTGGAGAAGCTGAAGAGCAA	F	60
		GGCACCTGCCCGAACTT	R	
ORX	AB056748	CCAGGAGCACGCTGAGAAG	F	67
		CCCATCTCAGTAAAAGCTCTTTGC	R	
ORXR1	AB110634	TGCGCTACCTCTGGAAGGA	F	58
		GCGATCAGCGCCCATTC	R	
ORXR2	XM_004945362	AAGTGCTGAAGCAACCATTGC	F	61
		AAGGCCACACTCTCCCTTCTG	R	
CRH	NM_001123031	TCAGCACCAGAGCCATCACA	F	74
		GCTCTATAAAAATAAAGAGGTGACATCAGA	R	
Ghrelin	AY303688	CACTCCTGCTCACATACAAGTTCA	F	75
		TCATATGTACACCTGTGGCAGAAA	R	
GHSR	NM_204394	GCACAAATCGGCAAGGAAA	F	61
		GTGACATCTCCCAGCAAATCC	R	
MC1R	NM_001031462	GCTCTGCCTCATTGGCTTCT	F	76
		TGCCAGCGCGAACATGT	R	
MC2R	NM_001031515	GCTGTTGGGCCCCCTTT	F	60
		AAGGGTTGTGTGGGCAAAAC	R	
MC3R	AB017137	GCCTCCCTTTACGTTCACATGT	F	59
		GCTGCGATGCGCTTCAC	R	
MC4R	NM_001031514	CCTCGGGAGGCTGCTATGA	F	62
		GATGCCCAGAGTCACAAACACTT	R	
MC5R	NM_001031015	GCCCTGCGTTACCACAACAT	F	63
		CCAAATGCATGCAATGATAAGC	R	
Ob-R	NM_204323	GCAAGACCCTCTCCCTTATCTCT	F	70
		TCTGTGAAAGCATCATCCTGATCT	R	
Adip	AY786316	ATGGACAAAAGGGAGACAAAGG	F	64
		TCCAGCACCCATATACCCAAA	R	
AdipR1	NM_001031027	CCGGGCAAATTCGACATC	F	58
		CCACCACGAGCACATGGA	R	
AdipR2	NM_001007854	TTGCCACTCGGAAGGTGTTT	F	60
		AGTGCAATGCCAGAATAATCCA	R	
Visfatin	NM_001030728	CCGGTAGCTGATCCAAACAAA	F	65
		CCAGCAGGTGTCCTATGCAA	R	
NPGL	AB909129	CCCTCAGTGCTGGGAATCC	F	61
		AGAAATGCGAGGCTTCCTCAT	R	
NPGM	XM_040665724.1	CACGGGCTGGTGGAAATG	F	65
		ATGAAGTCCCAGAGAGCAATGAC	R	
18S	AF173612	TCCCCTCCCGTTACTTGGAT	F	60
		GCGCTCGTCGGCATGTA	R	

*^†^Accession number refers to GenBank (National Center for Biotechnology Information—NCBI).*

*^§^F, forward; R, reverse.*

*NPY, neuropeptide Y; AgRP, agouti-related peptide; POMC, proopiomelanocortin; CART, cocaine and amphetamine regulated transcript; ORX, orexin; ORXR1, orexin receptor 1; ORXR2, orexin receptor 2; CRH, corticotropin releasing hormone; GHR, growth hormone receptor; GHSR, growth hormone secretagogue receptor; MC1R, melanocortin receptor 1; MC2R, melanocortin receptor 2; MC3R, melanocortin receptor 3; MC4R, melanocortin receptor 4; MC5R, melanocortin receptor 5; Ob-R, leptin receptor; Adip, adiponectin; AdipR1, adiponectin receptors 1; AdipR2, adiponectin receptors 2; NPGL, neurosecretory protein GL; NPGM, neurosecretory protein GM.*

### Statistical Analysis

Performance and mRNA expression data were analyzed through two-way ANOVA with interaction between the main factors, namely environmental temperature, and line. The pen (i.e., the replicate) and sampled animal were the experimental units for performance and mRNA expression analysis, respectively. The significance level was set at 0.05. Tukey’s honestly significant difference test was used as *post hoc*. These analyses were carried out in R (R Core Team, 2020).

## Results

### Heat Stress Differentially Depressed Feed Intake and Altered the Expression of Feeding-Related Hypothalamic Neuropeptides

**FIGURE 1:**
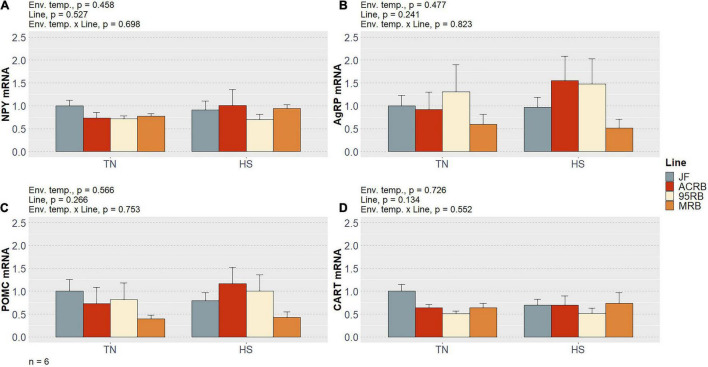
Effect of cyclic heat stress on the hypothalamic expression of NPY **(A)**, AgRP **(B)**, POMC **(C)**, and CART **(D)** in JF and modern broilers. The mRNA abundances were determined by qPCR using 2^–ΔΔ*Ct*^ method. JF-TN group was used as a calibrator. Data are mean ± SEM (*n* = 6 birds/group). HS, heat stress; TN, thermoneutral.

**FIGURE 2:**
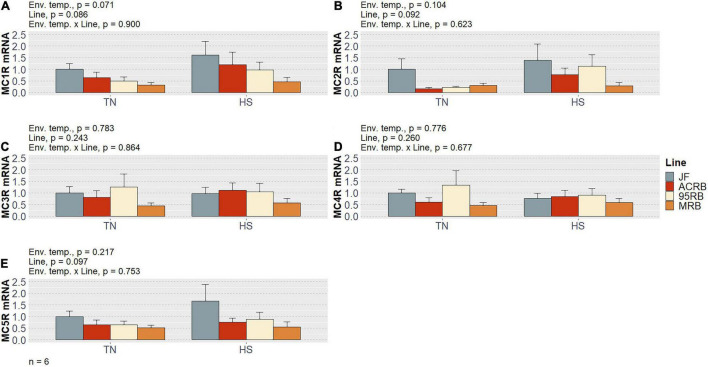
Effect of cyclic heat stress on the hypothalamic expression of the melanocortin receptor system in JF and modern broilers. The expression of MC1R **(A)**, MC2R **(B)**, MC3R **(C)**, MC4R **(D)**, and MC5R gene **(E)** was determined by qPCR using 2^–ΔΔ*Ct*^ method. JF-TN group was used as a calibrator. Data are mean ± SEM (*n* = 6 birds/group). HS, heat stress; TN, thermoneutral.

**FIGURE 3:**
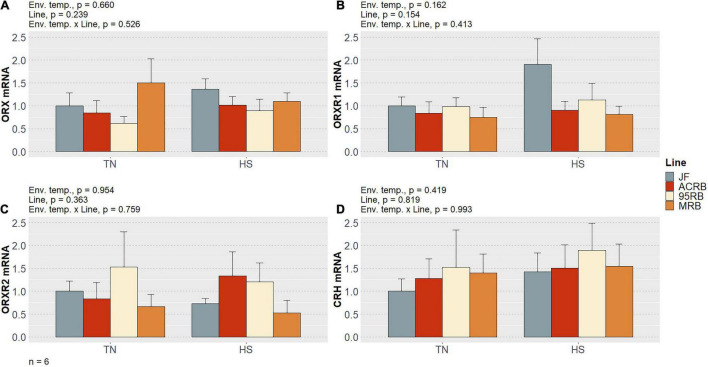
Effect of cyclic heat stress on the hypothalamic expression of the orexin system and CRH in JF and modern broilers. The expression of ORX **(A)**, ORXR1 **(B)**, ORXR2 **(C)**, and CRH gene **(D)** was determined by qPCR using 2^–ΔΔ*Ct*^ method. JF-TN group was used as a calibrator. Data are mean ± SEM (*n* = 6 birds/group). HS, heat stress; TN, thermoneutral.

**FIGURE 4:**
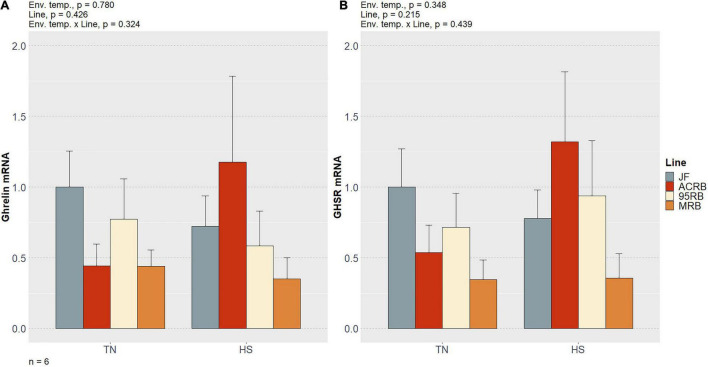
Effect of cyclic heat stress on the hypothalamic expression of ghrelin **(A)** and its receptor **(B)** in JF and modern broilers. The mRNA abundances were determined by qPCR using 2^–ΔΔ*Ct*^ method. JF-TN group was used as a calibrator. Data are mean ± SEM (*n* = 6 birds/group). HS, heat stress; TN, thermoneutral.

**FIGURE 5:**
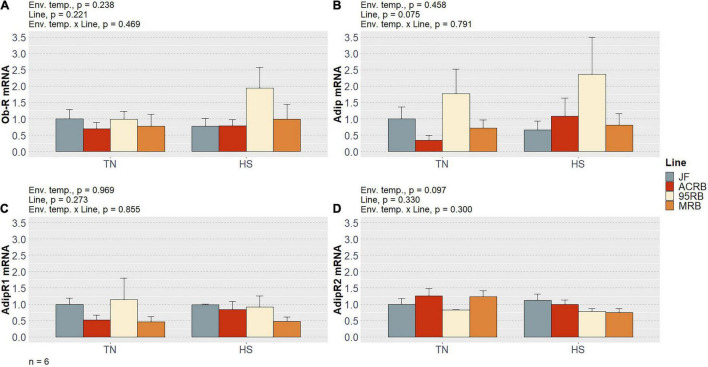
Effect of cyclic heat stress on the hypothalamic expression of adiponectin system and leptin receptor in JF and modern broilers. The expression of Ob-R **(A)**, Adip **(B)**, Adip-R1 **(C)**, and Adip-R2 gene **(D)** was determined by qPCR using 2^–ΔΔ*Ct*^ method. JF-TN group was used as a calibrator. Data are mean ± SEM (*n* = 6 birds/group). HS, heat stress; TN, thermoneutral.

**FIGURE 6:**
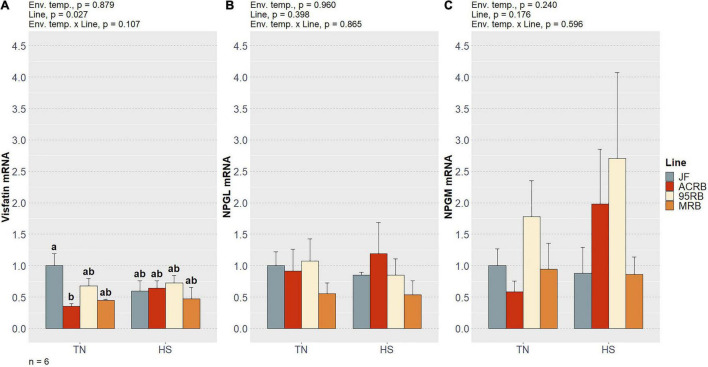
Effect of cyclic heat stress on the hypothalamic expression of visfatin **(A)**, NPGL **(B)**, and NPGM **(C)** in JF and modern broilers. The mRNA abundances were determined by qPCR using 2^–ΔΔ*Ct*^ method. JF-TN group was used as a calibrator. Data are mean ± SEM (*n* = 6 birds/group). HS, heat stress; TN, thermoneutral.

As previously shown by our group (Abdelli et al., 2021) and summarized in Table 3, chronic cyclic HS significantly decreased cumulative FI in both MRB and 95RB, but not in JF and ACRB. The highest cumulative FI was observed in MRB, followed by 95RB, and JF and ACRB regardless of the environmental conditions. These changes in FI resulted in similar modulation of BW, with highest weight observed in MRB, 95RB, and lowest weight in ACRB and JF (Table 3). At molecular level, neither environmental temperature nor line significantly influenced the hypothalamic expression of neuropeptide Y (NPY), agouti-related peptide (AgRP), proopiomelanocortin (POMC), and cocaine and amphetamine regulated transcript (CART) (Figure 1 and Supplementary Table 1). The mRNA expression of melanocortin receptors (MC1-5R) genes was not affected in a significant fashion, even though MC1R exhibited a line-dependent decreasing trend from JF to MRB under both TN and HS (*p* = 0.09) (Figure 2 and Supplementary Table 1). The expression of orexin (ORX), orexin receptors 1 and 2 (ORXR1-2), and corticotropin releasing hormone (CRH) genes appeared to be unaffected by environmental temperature and line (Figure 3 and Supplementary Table 1). Similarly, no significant differences were found for the expression of ghrelin and its receptor GHSR (Figure 4 and Supplementary Table 1). While the expression of leptin receptor (Ob-R) and adiponectin receptors 1 and 2 (AdipR1-2) remained unchanged, adiponectin (Adip) expression showed a distinct trend toward significance with 95RB exhibiting the highest mRNA level irrespective of the environmental temperature (*p* = 0.08) (Figure 5 and Supplementary Table 1). The environmental temperature did not affect the expression of visfatin and neurosecretory proteins GL and GM (NPGL and NPGM, respectively) (Figures 6B,C and Supplementary Table 1). However, under TN, JF had a greater mRNA abundance of visfatin than ACRB (*p* < 0.05) (Figure 6A and Supplementary Table 1).

**TABLE 3:** Effect of cyclic heat stress on cumulative feed intake (FI), final body weight (BW), and cumulative feed conversion ratio (FCR) (*n* = 3 replicate/group).

Trait	TN				HS				SEM	*p*-value		
	JF	ACRB	95RB	MRB	JF	ACRB	95RB	MRB		Env. temp.	Line	Env. temp. × Line
Cumulative FI (kg/bird)	1.98^e^	1.95^e^	5.96^c^	7.77^a^	1.99^e^	1.86*^e^*	5.40^d^	7.06^b^	0.09	< 0.001	< 0.001	0.002
Final BW (kg/bird)	0.88^e^	0.97^e^	3.19^c^	5.01^a^	0.86^e^	0.94^e^	2.95^d^	4.46*^b^*	0.04	< 0.001	< 0.001	< 0.001
Cumulative FCR	2.35^a^	2.08^b^	1.89^bc^	1.56^d^	2.40^a^	2.06^bc^	1.85^c^	1.60^d^	0.04	0.819	< 0.001	0.719

*Within a row, means with different superscripts are significantly different (p < 0.05). HS, heat stress; TN, thermoneutral.*

## Discussion

A 70-year genetic progress has been instrumental in making the poultry sector a cost-effective source of high-quality and affordable proteins worldwide. Intensive genetic selection programs have produced broiler chickens capable of efficiently converting feed into muscle mass (Clavijo and Flórez, 2018) with tremendously high growth rates and processing yields (Havenstein et al., 1994a,b, 2003a,b; Zuidhof et al., 2014; Tallentire et al., 2016). Results reported in our previously published papers (Orlowski et al., 2020; Abdelli et al., 2021) are consistent with the improvements in productive performance and slaughter traits ascribable to selection. By outperforming the other lines regardless of the environmental temperature, MRB corroborated the valuable outcomes of selection. Although they have become very efficient, modern broilers are facing unintended drawbacks caused by genetic selection.

In addition to welfare issues detailed by Hartcher and Lum (2020), current broilers are more sensitive to elevated ambient temperatures due to high metabolic rates (Cahaner and Leenstra, 1992; Yunis and Cahaner, 1999; Yahav, 2009). Even though selection has remodeled the broiler chicken body (Zuidhof et al., 2014), this homeothermic bird still employs the same thermoregulatory apparatus and strategies as its undomesticated forebear (Renaudeau et al., 2012; Rajaei-Sharifabadi et al., 2017; Rostagno, 2020). When exposed to HS, endothermic animals generally reduce feed consumption to limit the metabolic heat released by the gastrointestinal activities and use of nutrients (Baumgard and Rhoads, 2013).

As recently shown by Abdelli et al. (2021) and summarized here (Table 3), the significant drops in FI of 95RB and MRB are a clear reflection of a more pronounced HS susceptibility of hyper-selected broilers than their predecessors. Since FI is tightly controlled by the hypothalamic centers of hunger and satiety (Simpson et al., 2008; Waterson and Horvath, 2015; Timper and Brüning, 2017; Luquet et al., 2019) through a myriad of (an)orexigenic neuropeptides (Arora and Anubhuti, 2006; Furuse et al., 2007; Tachibana and Tsutsui, 2016; Delgado et al., 2017), we sought to determine the expression profile of FRHN in four chicken populations undergoing chronic cyclic HS.

NPY, AgRP, POMC, and CART represent the most renowned FRHN. NPY and AgRP are orexigenic peptides co-expressed by a subpopulation of neurons located in the infundibular nucleus (IN) of the avian hypothalamus (equivalent to the mammalian arcuate nucleus—ARC). Notably, NPY has been considered to be the most potent appetite-stimulating factor since the pioneering work of Stanley and Leibowitz (1985) dating back to mid-1980s. On the other hand, POMC and CART, produced by another subpopulation of IN neurons, act as anorexigenic or anorectic peptides (Delgado et al., 2017; Gaspar and Velloso, 2018). Since in this study no variation was detected for the expression of these major FHRN, it could conceivably be assumed that they are not linked to FI differences of the four lines exposed to either TN or HS.

POMC-derived ligands, collectively named melanocortins, bind to five melanocortin receptors (i.e., MC1-5R) to play their anorexigenic role (Tachibana and Tsutsui, 2016). In this study, MC1R showed a line-dependent trend under both environmental conditions, with a gradually decreasing mRNA abundance across the four lines. We have also previously reported an overexpression of melanocortin receptors in male quails selected for low feed efficiency (Blankenship et al., 2015). Future research on hypothalamic melanocortin system and its different expression in poor- and high-performing poultry is therefore warranted.

Orexin system, encompassing ORX and its receptors, was unaffected in our experimental conditions. While mammalian orexins—also known as hypocretins—have been shown to considerably stimulate appetite, the effect of orexins on chicken feeding behavior is still unclear and matter of debate (Furuse et al., 1999). Nonetheless, we have recently found that ORX system is central in both muscular and hepatic energy metabolism of avian species (Lassiter et al., 2015; Greene et al., 2020).

CRH, widely known for its involvement in stress response (Weninger et al., 1999), has been shown to possess a substantial anorectic effect in chicken (Furuse et al., 1997). Interestingly, Tachibana and Tsutsui (2016) posited that CRH can be a downstream mediator for the anorexigenic neural pathway in chicken brain. The environmental stressor applied in this study was expected to increase the hypothalamic expression of CRH, with a consequent CRH-mediated role in FI depression exhibited by 95RB and MRB subjected to HS. However, no differences were found in mRNA level of CRH.

Some neuropeptides can play divergent roles according to the animal class (Volkoff et al., 2005; Luquet et al., 2019). Ghrelin, a peripheral peptidergic hormone mainly released by the gastric mucosa (Arora and Anubhuti, 2006), is a clear example of these discrepancies (Tachibana and Tsutsui, 2016). Contrary to mammals and many fishes, ghrelin suppresses FI in chicken (Furuse et al., 2001; Tachibana and Tsutsui, 2016) and quails (Shousha et al., 2005) via a cascade of events discovered by Saito et al. (2005). They found out that the intracerebroventricular injection of ghrelin induces a CRH-dependent activation of the hypothalamic-pituitary-adrenal axis resulting in corticosterone secretion and, eventually, hyperactivity and anorexia in chicks. These researchers also confirmed earlier findings demonstrating that ghrelin is expressed in chicken brain (Kaiya et al., 2002). Moreover, at hypothalamic and pituitary level, ghrelin has been shown to bind to GHSR, a G-protein coupled receptor that modulates the release of growth hormone (Wren et al., 2000). In the present study, neither ghrelin nor GHSR hypothalamic expression exhibited significant line-dependent differences under both TN and HS.

Leptin receptor (Ob-R) has been shown to have a pivotal role in feeding control of chicken. Indeed, Lei et al. (2018) have lately demonstrated that the administration of anti-Ob-R antibodies promotes FI in growing chickens. We have previously examined the targets of leptin in chicken hypothalamus (Dridi et al., 2005) revealing, among other things, that it downregulates the expression of its own receptor. In this study, however, the between-line differences in Ob-R mRNA level were not significant under both environmental conditions.

When it comes to adipose tissue-derived peptides involved in regulation of feeding behavior and energy homeostasis, Adip and visfatin are also worth mentioning. In addition to adipocytes, Adip is expressed in several chicken tissues (Maddineni et al., 2005) while its receptors, viz. AdipR1-2, are abundantly present in the hypothalamus (Kadowaki et al., 2008). Kadowaki et al. (2008) thoroughly reviewed the appetite stimulation, longer-term fat modulation, and starvation signaling properties of Adip system, along with its antagonistic effect to leptin. Interestingly, we detected a tendency of 95RB to overexpress Adip regardless of the environmental temperature. It is difficult to interpret this result and further investigations are required to clarify the role of Adip system in hypothalamic FI modulation of chicken.

Visfatin, the second adipokine mentioned before, has been shown to have a wide array of physiological and pathophysiological functions (Dahl et al., 2012). In contrast to mammals, visfatin stimulates appetite in chicken (Cline et al., 2008). Furthermore, we have formerly established that visfatin is ubiquitously expressed in chicken and interconnected with numerous regulatory factors of energy balance (Ons et al., 2010). Here, the observed difference in visfatin expression between JF and ACRB provides further evidence to reasonably believe that visfatin modulates the feeding behavior of chicken. While the involvement of several FRHN in feeding regulation has been verified and their mechanism of action successfully elucidated, many others are still under study or have not even been identified (Parker and Bloom, 2012).

In this regard, two novel hypothalamic neuropeptides, called NPGL and NPGM, have been lately discovered in chicken (Ukena et al., 2014; Shikano et al., 2018). Shikano et al. (2017, 2018) found that NPGL is orexigenic, whereas NPGM has anorectic properties. Although we did not detect a connection between these new FRHN and the observed FI differences, additional studies on chicken NPGL and NPGM are strongly recommended.

There are several possible explanations for the lack of variations observed in this study. The first speculation is based on the hypothalamic integration of circadian information (Bechtold and Loudon, 2013; Delgado et al., 2017). The fact that birds have been fed prior to sampling and sacrificed at different times due to unavoidable operational needs may have remarkably impinged on the hypothalamus, resulting in a flattening of FRHN expression. A second reason might be related to the persistence of the environmental stressor: birds might have gradually acclimatized to the long-lasting exposure to high ambient temperature, thereby accommodating their physiological and hypothalamic response to chronic HS (Sykes and Fataftah, 1986; Yahav, 2009; Collier et al., 2019). Thirdly, the homogeneity showed by some major FRHN across the four chicken populations may partly be explained by stable hypothalamic pathways unaffected by evolution and genetic selection. Fourthly, we measured only mRNA abundances, while it is plausible that protein expression might have differently been affected. Lastly, the fact that we used the whole hypothalamus might have masked the mRNA expression in specific hypothalamic nuclei.

## Conclusion

In conclusion, this is the first assessment of the impacts of heat stress on feeding-related hypothalamic neuropeptides of chicken, with a valuable and informative comparison between the ancestor jungle fowl and three differently performing broiler lines.

## Data Availability Statement

The original contributions presented in the study are included in the article/Supplementary Material, further inquiries can be directed to the corresponding author/s.

## Ethics Statement

The present study was approved by the University of Arkansas Animal Care and Use Committee (protocols 18,083 and 16,084), and conducted in compliance with the guide for the care and use of laboratory animals of the National Institutes of Health.

## Author Contributions

SD conceived and designed the study. TT, EG, SO, and SD conducted the *in vivo* experiments. GB performed the molecular analyses, analyzed the data, and wrote the first draft of the manuscript. SD edited and corrected the manuscript with a critical review by all authors.

## Conflict of Interest

The authors declare that the research was conducted in the absence of any commercial or financial relationships that could be construed as a potential conflict of interest.

## Publisher’s Note

All claims expressed in this article are solely those of the authors and do not necessarily represent those of their affiliated organizations, or those of the publisher, the editors and the reviewers. Any product that may be evaluated in this article, or claim that may be made by its manufacturer, is not guaranteed or endorsed by the publisher.
