# Supplementary material for: Effect of Cyclic Heat Stress on Feeding-Related Hypothalamic Neuropeptides of Three Broiler Populations and Their Ancestor Jungle Fowl
Source: Front Physiol. 2021 Dec 23;12:809341. doi: 10.3389/fphys.2021.809341 (PMC8733626; doi:10.3389/fphys.2021.809341)
Supplement: Supplementary file 1 [file Table_1.DOCX]

**Table S1.** Effect of cyclic heat stress on the expression of feeding-related hypothalamic neuropeptides (FRHN) (*n* = 6 birds/group). The mRNA abundances were determined by qPCR using 2^-∆∆^*^C^*^T^ method. JF-TN group was used as a calibrator. Within a row, means with different superscripts are significantly different (*p* < 0.05). HS, heat stress; TN, thermoneutral.

| **FRHN** | **TN** | | | | **HS** | | | | **SEM** | ***p*-value** | | |  |
| --- | --- | --- | --- | --- | --- | --- | --- | --- | --- | --- | --- | --- | --- |
|  | JF | ACRB | 95RB | MRB | JF | ACRB | 95RB | MRB |  | Env. Temp. | Line | Env. Temp.  ×  Line |  |
| NPY | 1.00 | 0.74 | 0.71 | 0.77 | 0.91 | 1.00 | 0.70 | 0.94 | 0.16 | 0.458 | 0.527 | 0.698 |  |
| AgRP | 1.00 | 0.92 | 1.31 | 0.60 | 0.96 | 1.55 | 1.48 | 0.51 | 0.37 | 0.477 | 0.241 | 0.823 |  |
| POMC | 1.00 | 0.72 | 0.82 | 0.39 | 0.79 | 1.17 | 1.00 | 0.43 | 0.26 | 0.566 | 0.266 | 0.753 |  |
| CART | 1.00 | 0.63 | 0.51 | 0.64 | 0.69 | 0.70 | 0.52 | 0.74 | 0.14 | 0.726 | 0.134 | 0.552 |  |
| ORX | 1.00 | 0.85 | 0.62 | 1.50 | 1.37 | 1.02 | 0.89 | 1.09 | 0.26 | 0.660 | 0.239 | 0.526 |  |
| ORXR1 | 1.00 | 0.84 | 0.99 | 0.75 | 1.90 | 0.90 | 1.12 | 0.81 | 0.27 | 0.162 | 0.154 | 0.413 |  |
| ORXR2 | 1.00 | 0.83 | 1.53 | 0.66 | 0.72 | 1.33 | 1.20 | 0.53 | 0.37 | 0.954 | 0.363 | 0.759 |  |
| CRH | 1.00 | 1.28 | 1.52 | 1.40 | 1.42 | 1.51 | 1.89 | 1.55 | 0.49 | 0.419 | 0.819 | 0.993 |  |
| Ghrelin | 1.00 | 0.44 | 0.77 | 0.44 | 0.72 | 1.18 | 0.59 | 0.35 | 0.25 | 0.780 | 0.426 | 0.324 |  |
| GHSR | 1.00 | 0.54 | 0.72 | 0.35 | 0.78 | 1.32 | 0.94 | 0.36 | 0.26 | 0.348 | 0.215 | 0.439 |  |
| MC1R | 1.00 | 0.64 | 0.50 | 0.32 | 1.62 | 1.20 | 0.97 | 0.47 | 0.30 | 0.071 | 0.086 | 0.900 |  |
| MC2R | 1.00 | 0.16 | 0.22 | 0.30 | 1.38 | 0.77 | 1.13 | 0.28 | 0.29 | 0.104 | 0.092 | 0.623 |  |
| MC3R | 1.00 | 0.80 | 1.26 | 0.45 | 0.97 | 1.12 | 1.05 | 0.58 | 0.29 | 0.783 | 0.243 | 0.864 |  |
| MC4R | 1.00 | 0.60 | 1.33 | 0.46 | 0.76 | 0.84 | 0.91 | 0.58 | 0.26 | 0.776 | 0.260 | 0.677 |  |
| MC5R | 1.00 | 0.64 | 0.64 | 0.52 | 1.66 | 0.75 | 0.89 | 0.54 | 0.27 | 0.217 | 0.097 | 0.753 |  |
| Ob-R | 1.00 | 0.70 | 0.99 | 0.77 | 0.77 | 0.78 | 1.94 | 0.99 | 0.33 | 0.238 | 0.221 | 0.469 |  |
| Adip | 1.00 | 0.34 | 1.77 | 0.72 | 0.66 | 1.08 | 2.36 | 0.81 | 0.48 | 0.458 | 0.075 | 0.791 |  |
| AdipR1 | 1.00 | 0.52 | 1.15 | 0.46 | 0.98 | 0.83 | 0.91 | 0.48 | 0.24 | 0.969 | 0.273 | 0.855 |  |
| AdipR2 | 1.00 | 1.25 | 0.82 | 1.23 | 1.12 | 0.99 | 0.78 | 0.75 | 0.14 | 0.097 | 0.330 | 0.300 |  |
| Visfatin | 1.00 | 0.35^a^ | 0.68^b^ | 0.45^ab^ | 0.60^ab^ | 0.64^ab^ | 0.72^ab^ | 0.47^ab^ | 0.13 | 0.879 | 0.027 | 0.107 |  |
| NPGL | 1.00 | 0.91 | 1.07 | 0.55 | 0.85 | 1.19 | 0.85 | 0.54 | 0.27 | 0.960 | 0.398 | 0.865 |  |
| NPGM | 1.00 | 0.58 | 1.78 | 0.94 | 0.88 | 1.98 | 2.71 | 0.86 | 0.54 | 0.240 | 0.176 | 0.596 |  |

NPY, neuropeptide Y; AgRP, agouti-related peptide; POMC, proopiomelanocortin; CART, cocaine and amphetamine regulated transcript; ORX, orexin; ORXR1, orexin receptor 1; ORXR2, orexin receptor 2; CRH, corticotropin releasing hormone; GHR, growth hormone receptor; GHSR, growth hormone secretagogue receptor; MC1R, melanocortin receptor 1; MC2R, melanocortin receptor 2; MC3R, melanocortin receptor 3; MC4R, melanocortin receptor 4; MC5R, melanocortin receptor 5; Ob-R, leptin receptor; Adip, adiponectin; AdipR1, adiponectin receptors 1; AdipR2, adiponectin receptors 2; NPGL, neurosecretory protein GL; NPGM, neurosecretory protein GM.
